# Calculating and unconvinced: exploring psychological antecedents of HPV vaccination among Polish-speaking Facebook users

**DOI:** 10.3389/fpubh.2026.1854931

**Published:** 2026-09-14

**Authors:** Julia Siebielec, Karolina Sobeczek, Anna Kavouras, Filip Raciborski

**Affiliations:** 1 Department of Environmental Hazard Prevention, Allergology and Immunology, Medical University of Warsaw, Warsaw, Poland; 2 Faculty of Health Sciences, Medical University of Warsaw, Warsaw, Poland

**Keywords:** 5C model, Facebook, HPV, immunization, social media, vaccination behavior, vaccine hesitancy

## Abstract

**Introduction:**

In 2023 Poland launched a free, nationwide HPV vaccination program for children aged 12 and 13 years. This study aims to identify psychological drivers behind HPV vaccination-related behavior among Polish-speaking Facebook users and links between different categories of drivers.

**Methods:**

Entries published between 15 October 2020 and 15 October 2023 were categorized based on five antecedents of vaccination, using a scale created in accordance with the 5C short scale (consisting of categories: confidence, complacency, constraints, calculation, and collective responsibility) tailored to the Polish language.

**Results:**

The discourse on HPV vaccination was present throughout the study period, with a significant rise in popularity in 2023, accounting for 51% of all entries. Timing of surges in entries was corresponding to HPV vaccine-related decisions of various authorities and events of special interest happening in the country and abroad. 42% of all entries were assigned to 5C model categories. Factors influencing HPV vaccine hesitancy coexisted, with the Calculation and Confidence categories being the most prevalent (60% and 47% of coded entries, respectively). 30% of coded entries included words only from the Calculation category. The most common combination was Confidence and Calculation, with 13% of entries belonging to both categories.

**Conclusion:**

Psychological factors affecting HPV vaccination include most commonly individuals' extensive information-seeking efforts, susceptibility to conspiracy theories, and their trust levels in vaccines and government authorities. HPV vaccination educational campaigns should target Facebook users with consideration of their diverse needs and psychological profiles and prioritize the dissemination of reliable information in an easily comprehensible manner to ensure optimal reception of the content.

## 1 Introduction

Human papillomavirus (HPV) is the most common sexually transmitted infection (1), responsible for almost 5% of all cancers worldwide (2), and detectable in more than 99% of cervical cancers (3). According to the ICO/IARC Information Centre on HPV and Cancer, cervical cancer is the sixth most common cancer among women in Poland, with 3,862 cases and 2,137 deaths recorded in 2023 (4).

HPV vaccination can significantly reduce the incidence of cervical cancer (5). The World Health Organization (WHO) recommends vaccination for girls aged 9–14 years before the initiation of sexual activity, as well as for older females and boys (6). The Centers for Disease Control and Prevention (CDC) recommends vaccinating children aged 11–12 years, teens, and young adults (7). In Europe, three types of prophylactic HPV vaccines are available: 2-valent, 4-valent, and 9-valent (8).

The first-dose HPV vaccination coverage rate in women is estimated at 21% worldwide and 37% in the WHO European Region (9). In Poland, approximately 10–15% of the eligible population is vaccinated against HPV (10).

In 2008, voluntary HPV vaccination for girls aged 11–12 years was included in the national immunization schedule in Poland (11). These vaccinations were financed either out-of-pocket, or by local self-government units as part of health policy programs (11). In June 2023, a nationwide HPV vaccination program was introduced, offering free HPV vaccinations for girls and boys aged 12 and 13 years with 2-valent Cervarix and 9-valent Gardasil (12).

In September 2023, only about 111,000 adolescents were vaccinated (13, 14) out of 800,000 children born in 2011 and 2010, eligible to participate in the program in its first year (15). These results are worth taking into the account considering that the National Oncology Strategy aims to vaccinate at least 60% of adolescents by 2028 (16). Attitudes toward vaccination are associated with many determinants, such as gender, age, ethnicity, upbringing, educational level, income, area of residence, as well as religious and political preferences (17–21). Factors influencing HPV vaccination behavior include insufficient awareness and knowledge about the HPV vaccine and the possible consequences related to HPV infection, lack of trust in government, as well as in vaccination effectiveness and safety, fear of the vaccine's side effects, and belief in controversial narratives or unverified claims (18, 20, 22). Comprehending the underlying factors and motivations behind the delay in vaccine acceptance or refusal is crucial for devising an effective response strategy informed by this understanding. The lack of such a response can lead to a decline in vaccination coverage and a higher incidence of vaccine-preventable diseases (23–25).

In 2019, WHO declared vaccine hesitancy (VH) as one of the top 10 global health threats. Although VH is a global phenomenon described and studied in various ways (23, 26–28), the concept of VH and its definition are broad and lack clarity (29–31).

Models, frameworks and theories that were applied to study VH, behavior, decision-making, beliefs, and concerns include Health Belief Model (32), Theory of Planned Behavior (33), C models – 3C, 4C and 5C (29, 34–36), 5A model (37), COM-B model (38), social ecological models (39) and Behavioral and Social Drivers of Vaccination framework (30).

HPV-related discourse is present on social media, as these platforms are easily accessible for conducting discussions and information searches (40). Facebook is one of the most popular social media platforms in Poland, with 26.37 million real users in 2022 (41). Facebook is a valuable source of information regarding the attitude toward the HPV vaccine worldwide (42) and in Poland (43). Individuals in Poland who discuss HPV vaccination on Facebook are likely to reflect the wider population of parents or caregivers, who are the actual decision-makers for adolescent vaccinations.

This study aims to identify psychological drivers behind HPV vaccination-related behavior among Polish-speaking Facebook users and links between different categories of drivers.

## 2 Materials and methods

### 2.1 Data collection

Data for the analysis was collected using the SentiOne tool for internet and social media monitoring and listening (SentiOne, Gdansk, Poland) (44). The query included the following keywords: szczepi^*^ [vaccin] hpv, szczepi wirus^*^ brodawczaka ludzkiego [vaccine^*^ human papillomavirus], cervarix, gardasil (42). The asterisk “^*^” indicates the inclusion of different conjugation and declension endings of the word. The collected posts and comments (entries) were published between 15 October 2020 and 15 October 2023, spanning 36 months.

A total of 1,048,575 entries were collected during the SentiOne query. A two-part selection process was conducted. Firstly, the language tag of the entries was set to Polish; secondly, the source of the entries was limited to social media platforms. After two rounds of selection, the number of entries included in the analysis decreased to 8,006. The final selection was made during the preliminary quantitative analysis in Microsoft Excel (Microsoft Excel, Microsoft, Redmond) (45). Out of 8,006 entries, 7,768 (97%) contained text. The remaining 238 entries (3%) consisted of graphic material and were not included in further analysis analysis due to software limitations–used program cannot process text in the images. Entries selection workflow is presented in Figure 1.

**Figure 1:**
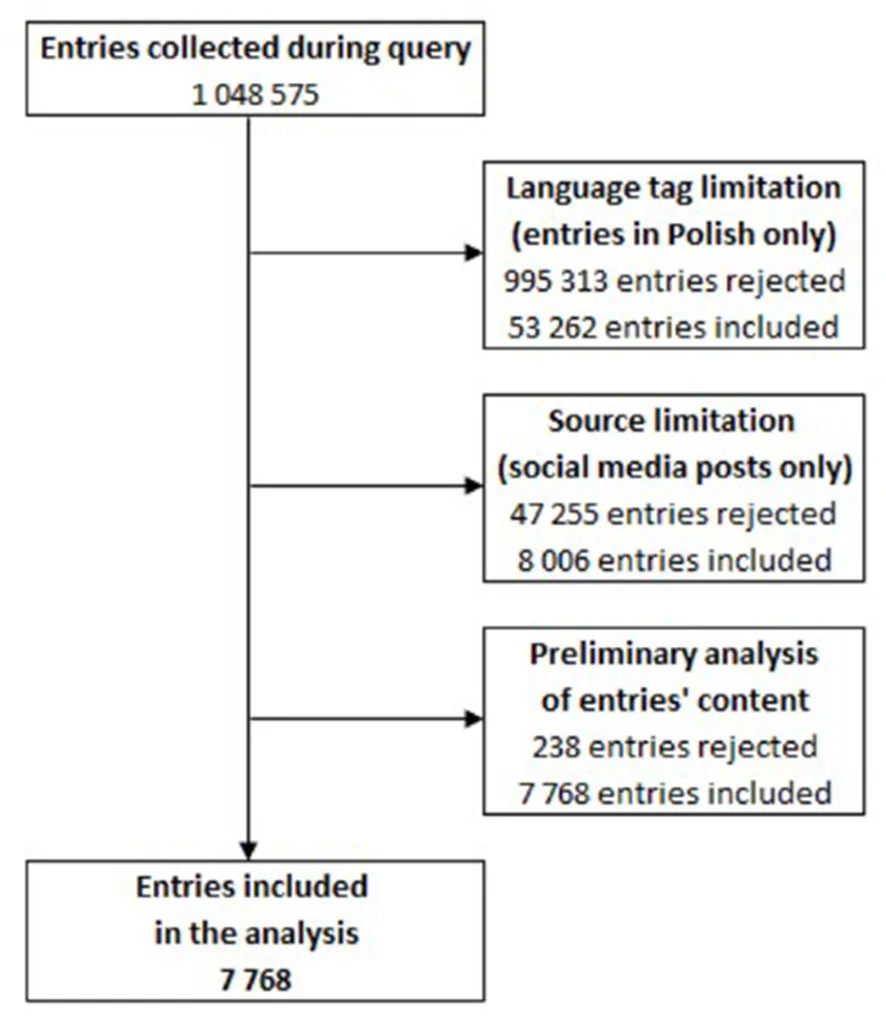
Entries selection workflow.

Following selection, entries were categorized according to the 5C short scale (35) using a coding framework and MaxQDa (MaxQDa, Verbi, Berlin) for qualitative data analysis (46). The 5C model was selected as it is one of the most widely established models, offering a structured approach to understanding the core areas that shape an individual's willingness and readiness to get vaccinated (47). The short scale was chosen to minimize bias associated with the assignment of a single coded word to multiple subscales.

### 2.2 Qualitative & quantitative analysis

The 5C model with the scale of psychological antecedents of vaccination is described in the context of VH but aims to assess the concepts of vaccination intention and behavior (31). It expands the 3C [Complacency, Confidence and Convenience (29)] and 4C [3C model with an addition of Calculation category (34)] models by adding and redefining categories of psychological antecedents. Antecedents are determinants or psychological factors existing prior to the decision to vaccinate that influence the vaccination decision process (36). The 5C antecedents are confidence, complacency, constraints, calculation, and collective responsibility.

- Confidence encompasses a range of issues related to trust, both in the safety and effectiveness of vaccines as well as in the system delivering, approving, and recommending vaccines. It is associated with belief in conspiracies, perception of risks, and knowledge about vaccination.
- Complacency is defined as not perceiving vaccine-preventable diseases as a high risk and is associated with perceived personal health status and invulnerability.
- Constraints are perceived barriers to vaccination, such as availability, affordability, and accessibility.
- Calculation is defined as a need for extensive information searching and personal risk-benefit calculations. It is associated with decision-making style and a tendency for superstitious beliefs.
- Collective responsibility relates to a perception of the common good and willingness to protect others (29, 34, 35).

The coding framework was developed by searching for English and Polish keywords in articles that examined vaccination behavior, intention, and hesitancy (48), considering Polish-language-specific pro- and anti-vaccination expressions as well as terms related to HPV vaccination.

The framework went through significant changes during our analysis: the initial version coded 2,764 entries (34.5%). To enhance the framework's sensitivity, a sample (*n* = 1,000) of uncoded entries was analyzed by identifying frequently used words and assessing their relevance in the context of HPV vaccination and the 5C model. New keywords were added to the appropriate categories, resulting in an updated version of the framework. After reviewing the framework, the number of coded entries increased by 37.9% to 3,758 (48.4% of all entries). To ensure the reliability of the results and coding accuracy, a random sample of entries (*n* = 400), selected using the RAND function in Excel, was analyzed. Manual review of entries assigned to each of the 5C model categories revealed that about 23.5% of the sample posts were categorized incorrectly. The framework was revised once more, with specific terms being eliminated to facilitate further refinements. After the adjustment, 87 entries (21.8% of the sample) were categorized incorrectly. Of those, 54 entries were unrelated to HPV vaccination discourse, instead addressing topics such as COVID-19 vaccinations, the Polish political situation, and natural medicine alternatives to vaccination. Among entries related to HPV vaccination discourse, only 33 out of 400 were categorized incorrectly (8.3% of the sample). The final version of the coding framework was used to categorize 3,256 (41.9%) of entries included in the coding process. The review process was conducted by one reviewer.

All versions of the framework together with English translation can be found in the Supplementary File. Added words are marked with *italics and* deleted words with strikethrough font.

The analysis of entries was conducted using the *autocode with dictionar*y function after transferring the coding framework into the *dictionary* database in MaxQDa. Textual analysis was performed. Embedded links within the entries were not verified, and the linked material was not included in the analysis. Entries without text content were excluded.

To verify the frequency of individual codes and combinations of codes, entries were analyzed using the *code configuration* option in MaxQDa.

Shapiro-Wilk test was performed to check the data distribution. 95% confidence intervals were calculated for each category's final framework result.

### 2.3 Material types

Three types of entries were analyzed within the research: comments (93.4%), posts (6.3%), and others (0.3%). The ‘other' category included links to external videos and texts. For every post published on the topic, there are 15 comments published as well.

## 3 Results

### 3.1 5C categories

Among the entries included in the analysis using the 5C scale, the most prevalent category was Calculation, with 1,956 entries (60.1% [95% CI: 58.4%; 61.8%]) containing keywords from this category, followed by Confidence with 1,542 entries (47.4% [95% CI: 45.7%; 49.1%]). The other three categories yielded substantially lower results: Complacency with 500 entries (15.4% [95% CI: 14.2%; 16.6%]), Collective Responsibility with 419 entries (12.9% [95% CI: 11.7%; 14.1%]), and Constraints with 409 entries (12.6% [95% CI: 11.5%; 13.7%]). The number of entries in each category is presented in Figure 2.

**Figure 2:**
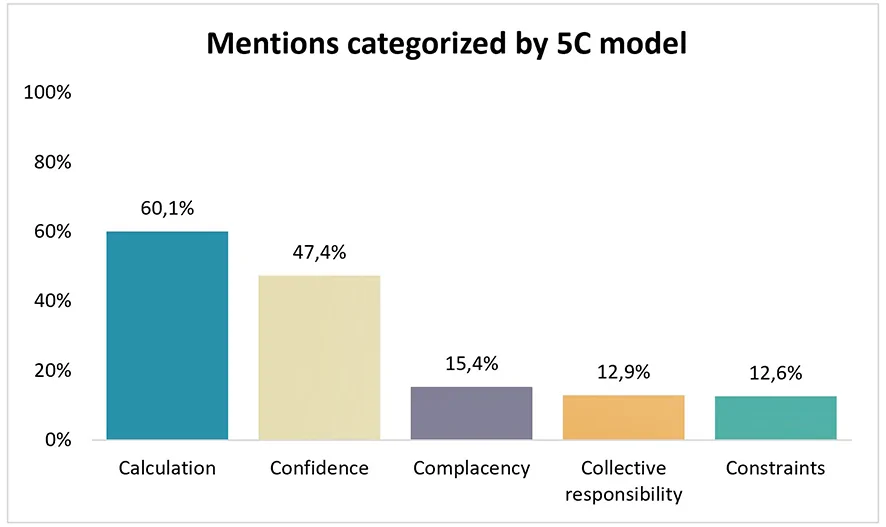
Percentage distribution of coded entries among 5C categories the percentage values next to the categories' names refer to entries containing keywords only from this category.

The results were found to be distributed normally (*p* = 0.057).

30% of entries contained keywords solely from the Calculation category, and 21.5% only from the Confidence category. The most common combination was Confidence and Calculation, comprising 13.3% of entries. The frequency of combinations of different codes is presented in Table 1. Only combinations registered in at least 1% of coded entries are included. Graphic presentation of codes combination frequency is presented in Figure 3.

**Table 1:** Categories' combination frequencies.

Codes	Percentage
Calculation	30.0%
Confidence	21.5%
Confidence + Calculation	13.3%
Collective responsibility	4.8%
Complacency	4.8%
Constraints	3.7%
Confidence + Complacency + Calculation	2.8%
Complacency + Calculation	2.3%
Confidence + Calculation + Collective responsibility	2.3%
Calculation + Constraints	2.2%
Calculation + Collective responsibility	1.9%
Confidence + Complacency	1.5%
Confidence + Calculation + Constraints	1.5%
Complacency + Calculation + Constraints	1.2%
Confidence + Constraints	1.2%
Confidence + Collective responsibility	1.0%

**Figure 3:**
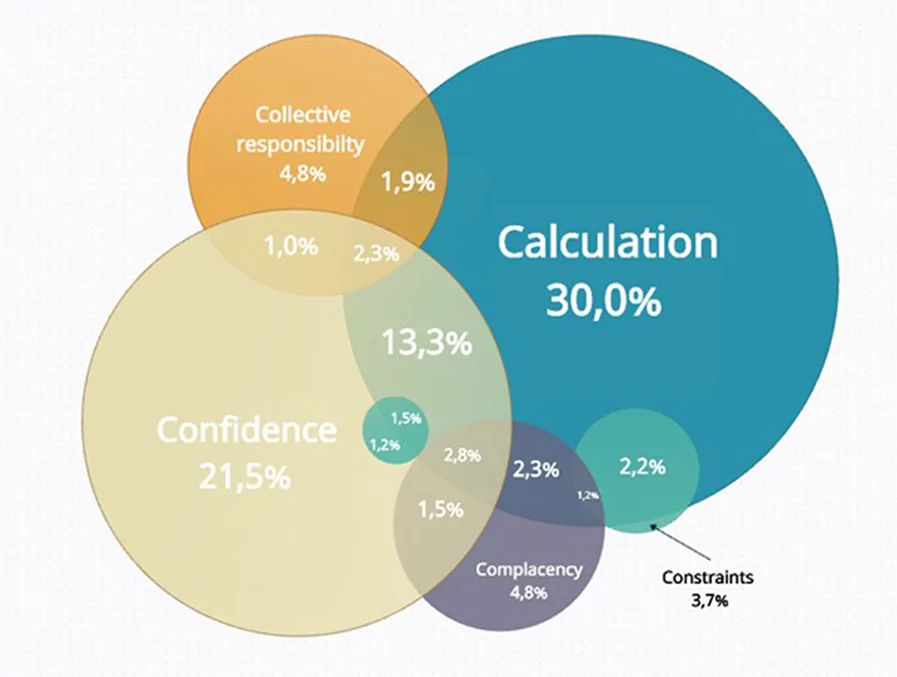
Venn diagram of categories combinations.

Entries assigned to the Calculation category included, on average, two keywords from that category. Confidence and Complacency categories included 1.8 and 1.5 keywords, respectively. Entries in the Constraints category contained 1.3 keywords per entry, while those in the Collective Responsibility category contained 1.2 words.

### 3.2 Discourse through the years

The topic of HPV vaccination has been present in online discourse over the past 4 years, with 1,511 (19.3%) entries published in 2020, 1,675 (21.3%) in 2021, 658 (8.2%) in 2022, and 4,162 (51.2%) in 2023. Approximately 8% of entries referenced controversies surrounding HPV vaccinations in other countries, primarily Denmark (6.1% of all entries) and Japan (1.6% of all entries). Approximately 3.2% of all entries consisted of copy-pasted spam responses, with a maximum repetition count of 15.

## 4 Discussion

### 4.1 Key results

The discourse on HPV vaccination persisted throughout the studied period, with a significant rise in popularity in 2023; surges of interest in the topic are correlated to events of high social interest linked to the vaccine. The discussion around the topic was mostly observed in comments sections. Factors influencing HPV vaccine behavior coexisted, as entries contained factors from multiple 5C model categories simultaneously. The Calculation and Confidence categories emerged as the most prevalent among the studied population. The analysis discovered a series of significant, sudden surges of published entries during the studied period; based on national media coverage and entries' contents, it is possible to assume that the peak in the number of entries in November 2020 may be attributed to the controversial decision of a Polish missionary bishop in Cameroon to prohibit HPV vaccinations for girls within his diocese (49). The significant increase in the number of entries observed in 2023 may be attributed to the launch of a free HPV vaccination programme by the Polish Ministry of Health. The peak of entry publications in 2023 occurred in June corresponding with the commencement of vaccinations under the programme. Timeline of entries publication thorough studied period is presented in Figure 4.

**Figure 4:**
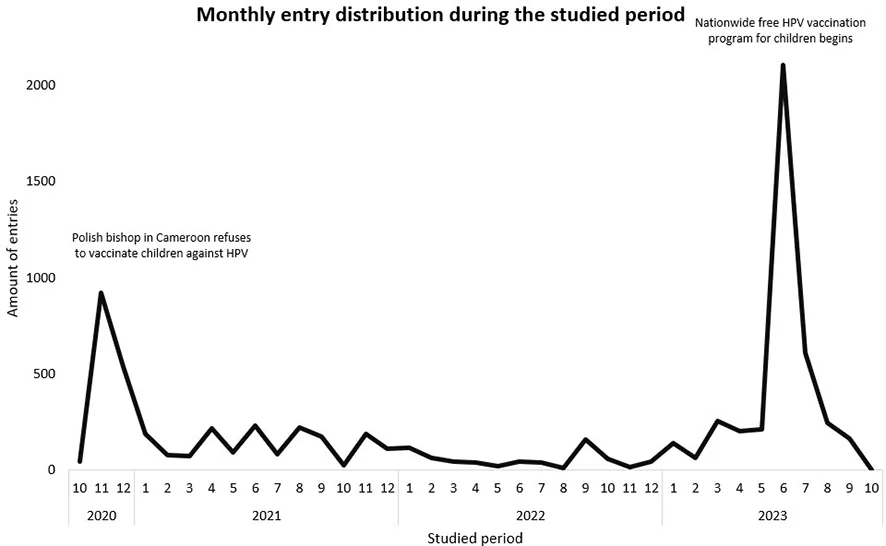
Timeline of entries publication through studied period.

### 4.2 Generalisability

HPV-related vaccination discourse is not a new subject among Polish-speaking Facebook users: other studies on the subject identified the theme occurring 2 years before this analysis (around 2018) (43). This fact as well as the prominence of the Calculation category within the studied population suggests that Polish-speaking Facebook users actively and deliberately sought HPV vaccination-related information on the topic (50). They likely aimed to encourage others to familiarize themselves with either facts or superstitious beliefs about HPV vaccination. Researchers identified knowledge as one of factors that influence vaccination attitude the most (51).

It is unclear what actual effect Confidence-category-related messaging has on its recipients; in a study researching messages related to vaccines on Scandinavian Twitter, the tweets classified into that category were not indicating a connection between willingness to seek information on the topic of HPV vaccines and willingness to vaccinate (52). The high ranking of the Confidence category may indicate that trust in HPV vaccines' safety and effectiveness, beliefs about its harms and benefits, trust in the Polish healthcare system, and conspiracy mentality play significant roles in HPV vaccination behavior among Polish Facebook users. Belief in vaccine conspiracies is especially dangerous as it is proven to be a major driver against vaccination (53). Maintaining trust in vaccine is crucial for positive vaccine behaviors. Concerns regarding the safety of HPV vaccines are a common reason to refuse vaccination (51). A more positive interpretation emerges from another analysis of the topic, which found that Polish-speaking Facebook users showed greater interest in sharing content expressing favorable views about the HPV vaccine than in posts conveying negative sentiment (43).

In the research conducted by the creators of the 5C scale, the Calculation category was found to be an important determinant of vaccine behavior and the most significant factor for US parents concerning MMR vaccination (35). However, the only statistically significant factor of HPV vaccine hesitancy among German parents is Confidence (35). A study analyzing Twitter vaccination-related entries (81% of which referred to the HPV vaccine) of Scandinavian users using the 5C scale revealed that Confidence was the most prevalent category, followed by Complacency (52).

Regarding COVID-19 vaccine hesitancy, differences in the importance of each category between studied populations are observed. Confidence and Collective Responsibility are indicated as the most important factors among non-pregnant individuals (54), Confidence among the United States population (55), and Convenience among Japanese men (54, 56). A study on Dutch, Belgian, and Portuguese students revealed Confidence and Collective Responsibility to be the two factors most strongly related to COVID-19 vaccination intention (57). A study on Hong Kong nurses also identified the Confidence category as the strongest factor associated with COVID-19 vaccination intention; a similar situation is observed regarding the influenza vaccine in the same population (58).

Disinformation present in vaccination-related online discourse in Japan (59) and Denmark (60) influenced the opinions of Polish social media users who propagated it. Symptoms mentioned in foreign discourse, such as fainting, chronic fatigue, and mobility issues, are characterized by the WHO as immunization stress-related responses and are not caused by the HPV vaccine itself (61).

### 4.3 Limitations

The main limitations of the study pertain to the collection and categorization of the material. Selecting appropriate keywords for the SentiOne query is crucial and may lead to inclusion bias. Due to the “context” section in the SentiOne query results, all comments responding to HPV vaccination-related posts were included, regardless of whether the comments themselves were related to HPV vaccination. Our coding framework may not encompass all the terms used to express opinions about HPV vaccination. This limitation arises from the specificity of the Polish language and the neologisms created by the anti-vaccine community during the COVID-19 pandemic to bypass social media platform censorship algorithms. Variations in the number of keywords in each category are predominantly due to the peculiarities of the Polish language. For instance, in the Constraints category, entries on the topic were similar and used specific, homogenous language. In the Confidence category, a broad range of expressions covered a spectrum of topics.

Another major limitation of presented analysis is the constraints of the algorithm used in the categorization process: the MaxQDa software cannot interpret more advanced human expressions such as humor or sarcasm. Analyzing data on the face value may have caused incorrect classification and such possibility should be taken into consideration while interpreting the results.

The manual review of framework's final version revealed 8.3% of entries were categorized incorrectly. Possibility of error of this scale should be taken into consideration while interpreting the results, especially since results of three categories are very close to one another (15.4% vs. 12.9% vs. 12.6%).

The generalisability of the results is limited due to the absence of demographic and socio-economic characteristics of Facebook users, which are crucial to fully understand the HPV vaccine-hesitancy determinants (62). It is also important to remember while the study examines the behavior of Facebook users, they are only a subset of entire population of people making decision related to vaccination. Described results may not be entirely accurate while scaling the discussion to the entire population.

This study focused on Facebook and included publicly available entries in text format. Discourse on other social media platforms, within private groups, or in video format may differ.

Additionally, the study has not taken into consideration the changes in popularity of individual categories and category combinations thorough the studied period. The influence of COVID-19 pandemic on studied discourse has also been not included in the analysis.

Another limitation of presented analysis is lack of inclusion of sentiment aspect. The focus of the analysis was to explore proportions of 5C categories in the HPV-related discourse, and the coding was prepared to categorize data in that regard. The results show the popularity of each category in the text, but not the represented sentiment. It is important to take this limitation into consideration while interpreting the results presented in this article.

## 5 Conclusion

Ongoing online discourse in Poland related to HPV vaccination gained significant popularity in 2023 following the introduction of the nationwide free HPV vaccination program. According to the 5C model, psychological factors affecting HPV vaccination include most commonly individuals' extensive information-seeking efforts, susceptibility to conspiracy theories, and their trust levels in vaccines and government authorities. HPV vaccination educational campaigns should target Facebook users with consideration of their diverse needs and psychological profiles by utilizing transparent institutional campaigns that openly reveal the risk-benefit balance of vaccination, in order to restore the lost “Confidence,” while simultaneously satisfying the prevailing and strong cognitive need for “Calculation” of the users to appropriately inform them about vaccination.

## Data Availability

The raw data supporting the conclusions of this article will be made available by the authors, without undue reservation.
