# Supplementary material for: Calculating and unconvinced: exploring psychological antecedents of HPV vaccination among Polish-speaking Facebook users
Source: Front Public Health. 2026 Sep 14;14:1854931. doi: 10.3389/fpubh.2026.1854931 (PMC13617130; doi:10.3389/fpubh.2026.1854931)
Supplement: Supplementary file 1 [file Data_Sheet_1.pdf]

Framework version 1.

Category	Coded word	Translation
**Confidence**	nop	undesirable post-vaccination reaction (abbreviation)
	odczyn	reaction
	szczepienn	vaccine-related
	wycofa*	recall
	sponsor*	sponsor
	konspir*	conspiracy
	zmow*	conspiracy (synonym)
	spisek	conspiracy (synonym)
	spisk*	conspiracy-related
	syf*	rubbish
	okalecz*	mulitate
	steryliz*	Sterilization
	płodn	Fertility
	powikła*	complications
	ufa	Trust
	profesjonaln*	Professional
	efekt	Effective
	bezpieczn	Safe
**Complacency**	naturaln*	natural
	odporn*	immunity
	bez wpływu	without influence
	nie warto	not worth [it]
	przebieg	course [of the disease]
	łagodn*	Mild
	zmien*	change
	zmień*	change [different grammatical ending]
	zdrow* organi*	healthy organism
	sensown*	Reasonable
	chron	protect/protected/protection
	ryzyk*	risk(s); risky
**Calculation**	sprawdz*	check
	artyku*	Article
	sprzeciw*	Oppose
	radz	Advise
	inform	information/inform/informed
	strzeg	warn/warning
	konsult	Consult
**Constraints**	miejsc*	placement [in a program]
	przychodni	Clinic
	infolin*	Hotline
	daleko	Far
	blisko	Close
	termin*	time slot
	kwot*	sum [can be used as a “price” in a context]
	pieniądz*	money
	dawk*	dose
	stać	afford
	cen*	Price
	dostęp	Available
	możliw	possible [to obtain]
	trudn*	Difficult
	łatw*	easy
	skomplikowan	Complicated
	osiągaln	achievable
	tan	cheap
	drog	expensive
**Collective responsibility**	wybór	choice
	wybor*	choice [different grammatical ending]
	prywatn*	private
	decyzj*	decision
	rodzic*	parents/parental
	narzuc*	[to] impose
	wtrąc*	interfere

	wola	will [of one own]
	łapy precz	“stay away from’ [colloquialism]
	nagonk*	battue
	indywid*	individual

Framework version 2.

Category	Coded word	Translation
**Confidence**	nop	undesirable post-vaccination reaction (abbreviation)
	odczyn	reaction
	szczepienn	vaccine-related
	wycofa*	[to] recall
	sponsor*	[to] sponsor
	konspir*	[to] conspire/conspiracy
	zmow*	conspiracy (synonym)
	spisek	conspiracy (synonym)
	spisk*	conspiracy-related
	syf*	rubbish
	okalecz*	[to] mulitate
	steryliz*	sterilization
	płodn	fertility
	powikła*	complications
	ufa	[to] trust
	profesjonaln*	professional
	efekt	effective
	bezpieczn	Safe
	truci*	_poison_
	doświadcz*	_[to] experiment (synonym)_
	eksperyment*	_[to] experiment_
	koncern*	_[pharmaceutical] concern(s)_
	lobb*	_[to] lobby/lobbied/lobbying_
	big pharm*	_Big Pharma_
	big farm*	_Big Pharma (phonetic writing)_
	wielka farmacja	_Big Pharma (Polish translation)_
	korpo*	_corporation_
	biznes	_Business_
	szpryc*	_vaccine (negative colloquialism)_
	szczyp*	_vaccine (negative colloquialism)_
	uboczn*	_side [effect]_
	szit*	_rubbish (phonetic writing of previously mentioned word “shit”)_
	kale*	_Cripple_
	rząd*	_government_
	nfz;	_National Health Fund (abbreviation)_
	dział*	_[to] work_
	truj*	_[to] poison_
	depopulacj*	_depopulation_
	oszu*	_[to] deceive; deceit_
	mafi*	_Mafia_
	trutk*	_poison (synonym)_
	*kłam*	_[to] lie_
	ściek*	_Sewage_
**Complacency**	naturaln*	Natural
	odporn*	Immunity
	bez wpływu	without influence
	nie warto	not worth [it]
	przebieg	course [of the disease]
	łagodn*	Mild
	zmien*	Change
	zmień*	change [different grammatical ending]
	zdrow* organi*	healthy organism
	sensown*	Reasonable
	chron	protect/protected/protection
	ryzyk*	Risk
	nie *trzeba	_there is no need_
	marnotraw*	_[to] waste_

	*celiba**	*Celibate*
	czystość	*purity*
	*bardziej bo**	*[someone is] more afraid [to]*
	*bardziej bał**	*[someone is] more afraid [to] (different grammatical ending)*
	*rozwiązł**	*[to be] promiscuous*
	*puszcz**	*[to be] promiscuous (synonym)*
	*zbędn**	*not necessary*
	**większ* ryzyk**	*[to] increase the risk*
**Calculation**	artyku*	Article
	sprawdz*	Check
	sprzeciw*	oppose
	radz	advise
	inform	information/inform/informed
	strzeg	warn/warning
	konsult	consult
	**czyta**	*[to] read*
	*zgłębi**	*[to] familiarize [oneself with sth]*
	*potwierdz**	*[to] confirm*
	*bada**	*[to] test*
	dane	*data*
	**informowan**	*[to] inform/be informed; information*
	*dokumen**	*[to] document*
	*film**	*film*
	**dowod**	*[to] proof*
	*publikacj**	*publication*
	**szuk**	*[to] seek*
	**pozna**	*[to] find out*
	*wywiad**	*interview*
	program	*programme*
	**świadom**	*[to] be aware; awareness*
	*eksper**	*expert(s); expertise*
	*stanowisk**	*statement*
	*powiąz**	*linked to*
	*kontrowers**	*controversy; controversial*
	*wpis**	*[blog] post*
	*materia**	*[scientific] material*
**Constraints**	przychodni	clinic
	miejsc*	placement [in a program]
	infolin*	hotline
	daleko	far
	blisko	close
	termin*	time slot
	kwot*	sum [can be used as a “price” in a context]
	pieniądz*	money
	dawk*	dose
	stać	afford
	cen*	price
	dostęp	available
	możliw	possible [to obtain]
	trudn*	Difficult
	łatw*	Easy
	skomplikowan	Complicated
	osiągaln	[un]reachable
	tan	Cheap
	drog	Expensive
	**polow**	*[to] hunt*
	*brak**	*[to] [be] lack(ing)*
**Collective responsibility**	wybor*	choice
	wybór	choice [different grammatical ending]
	prywatn*	private
	decyzj*	decision
	rodzic*	parents/parental
	narzuc*	[to] impose
	wtrąc*	interfere
	wola	will [of one own]
	łapy precz	“stay away from’ [colloquialism]

	nagonk*	battue
	indywid*	individual
	namawia*	_[to] urge_
	*musi*	_[to] force [someone]_
	parci*	_pressure (colloquialism)_
	za siebie	_for oneself_
	przymus*	_[under] pressure_

Framework version 3.

Category	Coded word	Translation
**Confidence**	nop	undesirable post-vaccination reaction (abbreviation)
	odczyn	reaction
	szczepienn	vaccine-related
	wycofa*	[to] recall
	sponsor*	[to] sponsor
	konspir*	[to] conspire/conspiracy
	zmow*	conspiracy (synonym)
	spisek	conspiracy (synonym)
	spisk*	conspiracy-related
	syf*	rubbish
	okalecz*	[to] mulitate
	steryliz*	sterilization
	płodn	fertility
	powikła*	complications
	ufa	[to] trust
	profesjonaln*	professional
	efekt	effective
	bezpieczn	Safe
	truci*	_poison_
	doświadcz*	_[to] experiment (synonym)_
	eksperyment*	_[to] experiment_
	koncern*	_[pharmaceutical] concern(s)_
	lobb*	_[to] lobby/lobbied/lobbying_
	big pharm*	_Big Pharma_
	big farm*	_Big Pharma (phonetic writing)_
	wielka farmacja	_Big Pharma (Polish translation)_
	korpo*	_corporation_
	biznes	_Business_
	szpryc*	_vaccine (negative colloquialism)_
	szczyp*	_vaccine (negative colloquialism)_
	uboczn*	_side [effect]_
	szit*	_rubbish (phonetic writing of previously mentioned word "shit")_
	kale*	_Cripple_
	~~_rząd*_~~	~~_government_~~
	nfz;	_National Health Fund (abbreviation)_
	dział*	_[to] work_

	truj*	[to] poison
	depopulacj*	depopulation
	~~oszu*~~	~~[to] deceive; deceit~~
	mafi*	Mafia
	trutk*	poison (synonym)
	kłam	[to] lie
	ściek*	Sewage
**Complacency**	~~naturaln*~~	~~Natural~~
	~~odporn*~~	~~Immunity~~
	bez wpływu	without influence
	nie warto	not worth [it]
	przebieg	course [of the disease]
	łagodn*	Mild
	~~zmien*~~	~~Change~~
	~~zmień*~~	~~change [different grammatical ending]~~
	zdrow* organi*	healthy organism
	sensown*	Reasonable
	chron	protect/protected/protection
	ryzyk*	Risk
	nie *trzeba	there is no need
	marnotraw*	[to] waste
	celiba*	Celibate
	czystość	purity
	bardziej bo*	[someone is] more afraid [to]
	bardziej bał*	[someone is] more afraid [to] (different grammatical ending)
	rozwiązł*	[to be] promiscuous
	puszcz*	[to be] promiscuous (synonym)
	zbędn*	not necessary
	większ ryzyk*	[to] increase the risk
	naturaln* odporn*	natural immunity
**Calculation**	artyku*	Article
	sprawdz*	Check
	sprzeciw*	oppose
	radz	advise
	inform	information/inform/informed
	~~*strzeg*~~	~~warn/warning~~
	konsult	consult
	czyta	[to] read
	zgłębi*	[to] familiarize [oneself with sth]
	potwierdz*	[to] confirm
	bada*	[to] test
	dane	data
	informowan	[to] inform/be informed; information
	dokumen*	[to] document

	*film**	*film*
	**dowod**	*[to] proof*
	*publikacj**	*publication*
	**szuk**	*[to] seek*
	**pozna**	*[to] find out*
	*wywiad**	*interview*
	program	*programme*
	**świadom**	*[to] be aware; awareness*
	*eksper**	*expert(s); expertise*
	~~*stanowisk**~~	~~*statement*~~
	~~*powiąz**~~	~~*linked to*~~
	*kontrowers**	*controversy; controversial*
	*wpis**	*[blog] post*
	*materia**	*[scientific] material*
**Constraints**	przychodni	clinic
	miejsc*	placement [in a program]
	infolin*	hotline
	daleko	far
	blisko	close
	termin*	time slot
	kwot*	sum [can be used as a "price" in a context]
	~~pieniądz*~~	~~money~~
	~~dawk*~~	~~dose~~
	stać	afford
	~~cen*~~	~~price~~
	dostęp	available
	~~*możliw*~~	~~possible [to obtain]~~
	~~trudn*~~	~~Difficult~~
	~~łatw*~~	~~Easy~~
	skomplikowan	Complicated
	osiągaln	[un]reachable
	~~*tan*~~	~~Cheap~~
	~~*drog*~~	~~Expensive~~
	**polow**	*[to] hunt*
	~~*brak**~~	~~*[to] [be] lack(ing)*~~
**Collective responsibility**	~~wybor*~~	~~choice~~
	~~wybór~~	~~choice [different grammatical ending]~~
	prywatn*	private
	decyzj*	decision
	rodzic*	parents/parental
	narzuc*	[to] impose
	wtrąc*	interfere
	wola	will [of one own]
	łapy precz	"stay away from' [colloquialism]

	nagonk*	battue
	~~indywid*~~	~~individual~~
	*namawia**	*[to] urge*
	~~**musi**~~	~~*[to] force [someone]*~~
	*parci**	*pressure (colloquialism)*
	za siebie	*for oneself*
	*przymus**	*[under] pressure*
